# The Case for Fungal Keratitis to Be Accepted as a Neglected Tropical Disease

**DOI:** 10.3390/jof8101047

**Published:** 2022-10-05

**Authors:** Lottie Brown, Guyguy Kamwiziku, Rita O. Oladele, Matthew J. Burton, N. Venkatesh Prajna, Thomas M. Leitman, David W. Denning

**Affiliations:** 1 Manchester Fungal Infection Group, Faculty of Biology, Medicine and Health, University of Manchester, Manchester M13 9PL, UK; 2 Kinshasa University Hospital, M8R4+CF3, Kinshasa P.O. Box 8842, Democratic Republic of the Congo; 3 Department of Medical Microbiology and Parasitology, College of Medicine, University of Lagos, Lagos 101017 , Nigeria; 4 International Centre for Eye Health, London School of Hygiene and Tropical Medicine, London WC1E 7HT, UK; 5 Aravind Eye Hospitals and Postgraduate Institute of Ophthalmology, Madurai 625020, Tamil Nadu, India; 6 Departments of Ophthalmology, Epidemiology & Biostatistics, University of California, San Francisco, CA 94143, USA; 7 Global Action for Fungal Infections, Rue Le Corbusier 12, 1208 Geneva, Switzerland

**Keywords:** fungal keratitis, neglected tropical diseases, blindness

## Abstract

Amongst the treatable cause of blindness among young people, fungal keratitis ranks high. There are an estimated 1,051,787 to 1,480,916 eyes affected annually, with 8–11% of patients having to have the eye removed. Diagnosis requires a corneal scraping, direct microscopy and fungal culture with a large number of airborne fungi implicated. Treatment involves the intensive application of antifungal eye drops, preferably natamycin, often combined with surgery. In low-resource settings, inappropriate corticosteroid eye drops, ineffective antibacterial therapy, diagnostic delay or no diagnosis all contribute to poor ocular outcomes with blindness (unilateral or bilateral) common. Modern detailed guidelines on fungal keratitis diagnosis and management are lacking. Here, we argue that fungal keratitis should be included as a neglected tropical disease, which would facilitate greater awareness of the condition, improved diagnostic capability, and access to affordable antifungal eye medicine.

## 1. Introduction

Since the early 2000s, a total of twenty neglected tropical diseases (NTDs) have been identified by the World Health Organization (WHO). This diverse group of infectious diseases have a profound health and socioeconomic burden on impoverished populations across the globe and are recognised as being both a consequence and driver of poverty. Of the twenty NTDs identified by the WHO, there are two which affect the eye and cause blindness: trachoma and onchocerciasis [1,2]. In 2019, a proposal was made to add infectious corneal ulcers to the list of NTDs, but these calls went unheeded [3].

**Figure 1:**
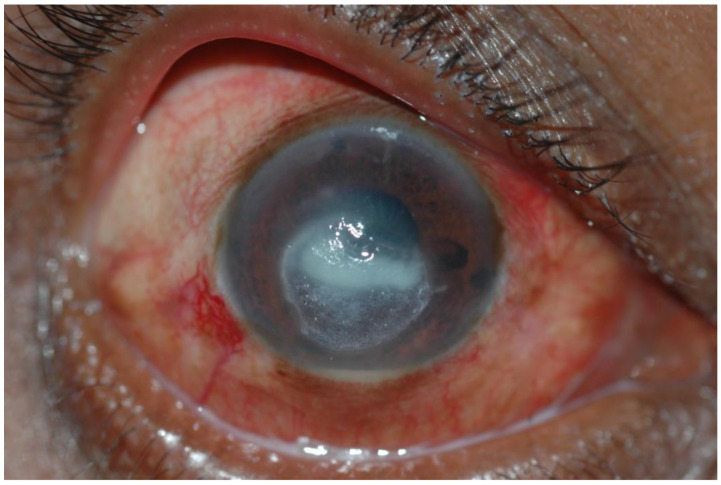
Corneal ulcer caused by *Fusarium* spp. at first presentation. Source: Mycotic Ulcer Treatment Trial, Aravind Eye Hospital, Tamil Nadu, India.

**Figure 2:**
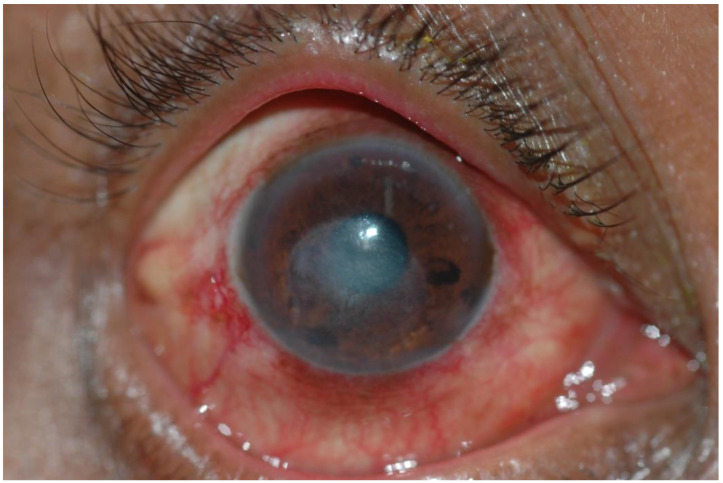
Corneal ulcer caused by *Fusarium* spp. 1 month later. Source: Mycotic Ulcer Treatment Trial, Aravind Eye Hospital, Tamil Nadu, India.

**Figure 3:**
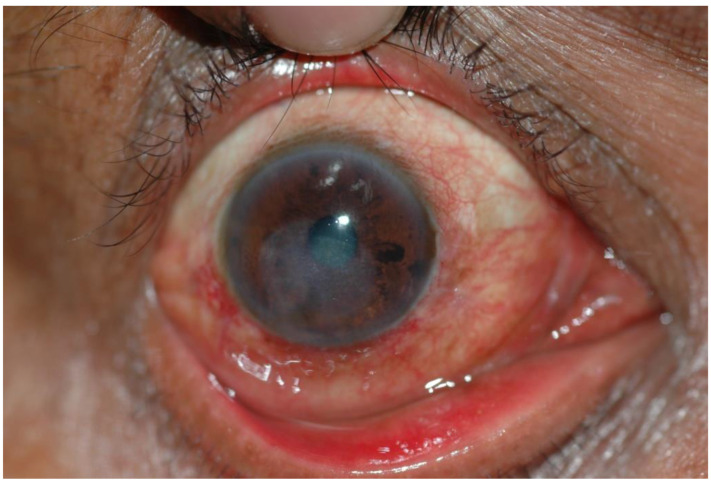
Corneal ulcer caused by *Fusarium* spp. at 3 months. Source: Mycotic Ulcer Treatment Trial, Aravind Eye Hospital, Tamil Nadu, India.

Fungal keratitis is a severe eye infection involving the cornea. It is an ocular emergency. The condition is most prevalent in tropical and subtropical regions and has been documented to account for between 37.7% and 81.5% of all culture-positive corneal infections in these climates, where it is a major cause of blindness, visual impairment and eye loss [4,5]. Over 100 causative agents have been identified, but the vast majority of infections are caused by filamentous fungi such as *Fusarium* spp. and *Aspergillus* spp. [6,7] (Figure 1, Figure 2 and Figure 3). Prompt diagnosis and treatment can help to preserve vision. However, presentation is often delayed, leading to irreversible blindness and loss of the eye through a perforation of the globe or endophthalmitis [8]. Because of the unilateral nature of this disease, it is often underreported and given little programmatic attention. Corneal blindness is an important cause of blindness globally, yet fungal keratitis is often sidelined [9]. Guidelines for suspicion, diagnosis and management have been issued by the SE Asian office of the WHO (SEARO) in 2004 and summary guidance by the America Academy of Ophthalmology in 2014 [10,11]. Here, we argue that the WHO and other agencies should recognise fungal keratitis as an NTD.

## 2. Epidemiology and Association with Poverty

A recent systematic review estimated that between 1 and 1.4 million new cases of fungal keratitis occur each year. The estimated annual incidence ranged from 73 per 100,000 in South Asia to just 0.02 per 100,000 in Europe [4]. In four large case series from Pakistan [12], East Africa [8], Germany [13] and Thailand [14], 8–11.5% of patients required eviscerations, which represents an annual loss of 84,000–167,000 eyes. Using outcome data from the Pakistan study for low-income and middle-income countries, it is predicted that over 600,000 eyes will go blind because of fungal keratitis each year [12].

Several of the risk factors for fungal keratitis and development of sight-threatening complications are closely associated with poverty. As most cases occur secondary to minor ocular trauma, sufferers are often young, healthy agricultural or outdoor workers living in tropical or subtropical regions who are unfortunate enough to experience an injury from organic or vegetative matter such as during harvesting. Traumatising agents from a variety of plant and animal sources have been recorded [8,15]. Immunosuppressive conditions including HIV/AIDS are thought to predispose individuals to the disease [8]. Fungal keratitis is more common in males [4]. Contact lens wearers are also at increased risk of developing the condition, attributed to inadequate contact lens hygiene practices and contaminated cleaning solution use [16]. Poor sanitation may contribute.

Individuals in resource-limited settings are more likely to present late, with deep and extensive corneal ulceration, irreversible visual loss, scarring, perforation and endophthalmitis (Figure 1, Figure 2 and Figure 3). A study from East Africa found a median delay of 14 days between the onset of symptoms and presentation to the hospital, and this extended to 21 days if another facility was visited first. Inadequate or inappropriate initial treatment was initiated in 64% of cases [8]. A similar delay was reported among Ugandan patients [17]. Despite the addition of natamycin eye drops to the WHO Essential Medicines List (EML), antifungal eye drops are scarcely available outside of tertiary centres [6,18,19,20]. Individuals from impoverished, rural communities may struggle to access appropriate care due to the cost of treatment, loss of earnings and often long distances to travel to tertiary eye hospitals. Initial treatment at pharmacies or primary healthcare centres may be unhelpful, and in some cases harmful if narrow-spectrum antibiotics or topical steroids are administered [21]. The use of traditional eye remedies, which are often plant-based and non-sterile, may introduce additional infection [17]. When compared to patients with bacterial keratitis, patients with fungal keratitis incur significantly more costs on medications, irrespective of whether they heal with successful medical treatment or require surgery. The prolonged duration of treatment and the high costs of antifungal medications account for the significant economic burden of fungal keratitis [22].

The spectrum of pathogens implicated in fungal keratitis is extremely diverse. A 2019 systematic review yielded 393 species of fungi from 169 genera as causative agents [23]. This is a significant increase from the 144 species in 92 genera identified in 2012 [24]. It is unclear whether this change is due to an actual shift in epidemiology or increased reporting. Aetiology differs depending on geographical location. Filamentous fungi are responsible for the vast majority of infections in tropical and subtropical climates whereas yeasts (mainly *Candida* spp.) are most commonly identified as the causative agent in temperate infections. *Candida* keratitis usually occurs secondary to ocular defects, recent ophthalmic surgery and immunosuppression including due to systemic diseases such as HIV/AIDS and diabetes [6,7].

## 3. Public Health Consequences

Fungal keratitis disproportionately affects the working-age population living in rural regions. It often causes unilateral blindness in these individuals, and this is a significant ophthalmic public health problem, but not captured in global vision impairment statistics [9]. The burden of disability for this condition is huge, and for the individual, a poor visual and cosmetic outcome is detrimental to their quality of life. A recent case–control study from Uganda found that quality of life in individuals with microbial keratitis is severely reduced in the acute phase [25]. Despite improving with treatment and healing, quality of life remained significantly reduced compared to controls, even with the restoration of normal vision [25]. Unilateral blindness may increase the risk of subsequent work injuries. Sufferers may have difficulty finding and maintaining employment because of this. In many low-income countries, there is little legislation to protect disabled workers from unfair prejudice and so affected individuals may experience unemployment and poverty. Furthermore, the high risk of corneal opacity and eye loss has serious implications on an individual’s quality of life. Negative body image, low self-esteem, and depression may ensue. Disfiguration may also lead to stigma and discrimination and reduce the chances of finding employment or a marital partner and can disproportionately affect women. Affected individuals may become socially isolated and this, in turn, would impact a sufferer’s family. For the wider community, fungal keratitis has been called a “social and economic disaster” [26]. This is because the exposed population are predominantly economically productive young adults. By causing disease and disability in these individuals, fungal keratitis reduces productivity and reinforces the cycle of poverty. More work must be conducted to investigate the burden of disability and determine the quality-adjusted life years (QALYs) and disability-adjusted life years (DALYs) lost due to fungal keratitis.

## 4. Diagnosis

In contrast to bacterial keratitis, the symptoms of FK are often disproportionately less severe than might be expected considering the size of the ulcer. This may be one of the reasons why patients often present late to treatment centres, commonly with an advanced fungal corneal ulcer. Timely diagnosis of fungal keratitis can prevent irreversible corneal destruction and drastically improve the chances of complete recovery [5,6,8]. The gold standard for the diagnosis of fungal keratitis is the direct visualisation of fungal hyphae by microscopy of smears or culture of corneal scrapings. The technical skill to be able to perform corneal scraping is not uniform, especially in low- and middle-income countries. Global Action For Fungal Infection (GAFFI) has been conducting a survey of this capability in Africa, with respondents from all countries with a population over 1 million. Corneal scraping is not undertaken in any government or NGO-funded healthcare facility in 15 countries. In another eight countries, it is performed only rarely. Part of this limitation relates to the lack of ophthalmologists and partly to the equipment and skill required to perform the procedure and process the specimen.

The following set of smears are recommended: a Gram stain, a wet preparation (using potassium hydroxide or lactophenol cotton blue) and a specialised fungal stain (Giemsa, periodic acid Schiff, Gomori methenamine silver stain or calcofluor white) [6]. This diagnostic modality is inexpensive, relatively simple and yields results rapidly, which renders it suitable in low resource settings [7]. The sensitivity of microscopy has been reported to be 61–94% using potassium hydroxide, 85% using lactophenol blue and 36–50% using a traditional Gram stain [27]. If a fluorescence microscope is available, Calcofluor white (which highlights fungal cell walls) may be more sensitive than conventional microscopy, depending on the experience of the microscopist [28]. Calcofluor white is said to be a mainstay of diagnosis, and when combined with potassium hydroxide stains, sensitivity has been shown to rise to 98.3% [29]. In some rural areas, even basic technology such as light microscopy is not routinely available. Novel microscopy tools, such as a Foldscope, an origami-based microscope which can be assembled from a flat sheet of paper in under 10 minutes and costs less than USD 1, may provide an alternative and reduce delays to diagnosis and appropriate treatment. The Foldscope may be attached to smartphone by magnets, allowing for image capture. The tool has previously been explored for diagnosis of parasitic helminth infections and cervical smear cytology with high sensitivity. A small study comparing smartphone-mounted Foldscope to conventional light microscopy on 60 corneal scrapings found reasonable sensitivity (0.72) and high specificity (0.92) [30]. Importantly, the performance of novel tools such as Foldscope relies on experienced and skilled operators, who may be confined to major cities. However, with internet transfer, clinicians and laboratory personnel in specialist eye centres could review captured images and advise on likely diagnoses. A free online course is available in multiple languages to upskill laboratory workers and physicians in direct microscopy (www.microfungi.net, accessed on 4 October 2022).

To differentiate between species of fungi, smears should be performed alongside culture. Fungal growth generally requires 48–72 h, but some species may take longer to grow (up to 14–35 days) which may delay the identification of genera and species. While awaiting culture results, empirical treatment is given based on clinical suspicion and smear stain. In a recent systematic review, 40% of 34,275 samples were culture-negative for fungi, despite high suspicion that the correct diagnosis was fungal keratitis [4]. Bacterial co-infection is common, with between 6 and 40% of culture-positive cases of fungal keratitis yielding bacterial growth [16,31,32,33]. Keratitis caused by *Pythium insidiosum* is a diagnostic challenge as it presents similarly to fungal keratitis and oomycetes closely resemble fungal filaments on microscopy. A recent study identified potassium hydroxide and calcofluor white mount as the most useful methods to differentiate between fungal and *Pythium* keratitis, with high sensitivity and specificity [34]. Due to the diversity of fungi cultured from cases of fungal keratitis, high levels of expertise are required for the precise identification of the fungal genera and species isolated.

The significant limitations of traditional microbiological techniques have led to the development of new diagnostic tools such as in vivo confocal microscopy (IVCM) of the cornea, anterior segment optical coherence tomography (AS-OCT) and polymerase chain reaction (PCR) [7]. IVCM is a non-invasive technique that enables real-time identification of the causative pathogen in microbial keratitis, including filamentous fungal elements with a high sensitivity and specificity, but its suitability in a low-resource setting is limited by the need for a relatively expensive device and availability of operators who are trained in interpreting IVCM images [35]. AS-OCT is another non-invasive diagnostic modality which has been evaluated in fungal keratitis. The advantage of AS-OCT is its ability to ascertain the precise depth of infiltrations and extent of corneal oedema which cannot be characterised by slit-lamp or IVCM. Differentiation between fungal and other types of microbial keratitis is possible due to the characteristic morphology of mycotic ulcers, but the main appeal of AS-OCT is for monitoring disease and response to treatment [36]. Global access to CT scanners is extremely limited (there are fewer than one CT scanners per 1 million inhabitants in most LMICs), so it is doubtful AS-OCT will significantly improve diagnosis and treatment monitoring of in high-risk settings [37]. A variety of PCR assays have been developed with the aim to provide rapid diagnosis using only small amounts of sample. Some assays are non-specific and only confirm the presence of fungi using panfungal primers based on conserved sequences. Multiplex assays can provide species identification but are usually limited to the most common aetiologies, which may be problematic given the large spectrum of fungi implicated in fungal keratitis [7]. Due to test complexity and the high risk of environmental contamination, PCR analysis should be conducted in diagnostic laboratories, the availability of which is limited to specialized, tertiary centres in high-resource settings.

There is currently no point of care test for fungal keratitis and this remains a major obstacle in improving health outcomes for the condition. Artificial intelligence (AI) based on deep learning techniques has demonstrated promising performance in detecting microbial keratitis and differentiating between fungal, bacterial and viral causes when using slit-lamp or smartphone images, outperforming even specialist clinicians [38]. The utilisation of AI could overcome the need for highly trained operators and specialized equipment, which limits the use of many of the other diagnostic alternatives in low-resource settings. A number of recent studies indicate sensitivity and specificity between 70 and 100% when using a slit lamp or smartphone images alone [39,40,41,42]. So far, AI research in fungal keratitis has focused on image-based data, but diagnostic accuracy may improve further with the integration of clinical and epidemiological data into learning models. AI technology may complement and improve sensitivity of IVCM and OCT, but equipment costs and availability still limit their use to high resource settings [43].

## 5. Treatment

Guidelines for the management of fungal keratitis were published in 2004 by the WHO Regional Office for South East Asia [10] and summary guidance by the American Academy of Ophthalmology in 2014 [11]. Medical management of fungal keratitis is always the first line, but various surgical procedures may also be required if complications ensue. Medical therapy includes specific antifungal drugs (topical or systemic) and non-specific, supportive methods (such as cycloplegics). Treatment responses to topical antifungal therapy are reasonable, with 75% of corneas not severely affected and 60% of those severely affected being effectively managed by topical 5% natamycin, now listed by WHO as an essential medicine [6,18]. Natamycin has a broad spectrum of antifungal activity and is active a low concentration. Outcomes are better if antifungal therapy is administered within the first 24 h of presentation [14]. Fungal keratitis usually responds to treatment slowly over several weeks. Less effective alternative therapies include voriconazole 1% eye drops, chlorhexidine 0.2% and systemic therapy with itraconazole or voriconazole [7,44,45,46]. Randomized controlled trials (RCT) have shown that natamycin was superior in terms of visual improvement and prevention of complications and that voriconazole should not be recommended as a monotherapy for filamentous fungal keratitis [47]. An RCT has also shown that adjuvant oral voriconazole did not have any added benefit in the rate of perforation and/or need for therapeutic keratoplasty, visual acuity, scar size or rate of re-epithelialization [45]. There were significantly more adverse events in the oral voriconazole group, including elevations in liver enzymes and visual disturbances, than patients in the placebo group [45].

A small number of cohort studies have examined the impact of corticosteroids on outcomes in fungal keratitis. Topical steroid therapy does not improve visual outcomes in exogenous fungal endophthalmitis as Cho et al. demonstrated in a retrospective cohort study of 83 patients diagnosed in South Korea [48]. The 30 patients who were empirically treated with corticosteroids had worse visual outcomes, more surgical inventions, and a higher rate of overall treatment failure. On the other hand, corticosteroids may have value in damping down immune rejection after penetrating or lamellar keratoplasty for fungal keratitis [49]. In their 244-eye study, 0.02% fluorometholone eye drops given twice daily were started one week after keratoplasty, which and increased to four times daily if there was no sign of fungal recurrence. Fungal recurrence was seen in 1.2% of eyes and 8 of 118 eyes with penetrating keratoplasty had allograft rejection, from 2 to 6 months post-procedure, which was more aggressively treated.

To prevent recurrence, antifungal therapy should be maintained for at least 6 weeks, regardless of negative corneal scrapings. Such long treatment regimens may affect compliance, particularly where individuals are required to pay for medication. Moreover, it is recommended that natamycin (and other topical antifungals) are applied hourly around the clock in the first few days of treatment. The practicality of these regimens is also likely to affect compliance [6]. Several randomized controlled trials investigating optimal antifungal regimens and other aspects of management have been performed, but more data are required. The development of topical formulations with a longer half-life is of high importance. In spite of appropriate treatment, fungal keratitis has higher odds of perforation and longer healing time than bacterial keratitis [50]. Newer adjuvant treatment modalities such as intrastromal voriconazole, corneal collagen cross-linking and Rosebengal photodynamic therapy have been tried with varying outcomes [51,52,53].

Ophthalmic surgery is sometimes required in patients who fail to respond to medical therapy or where there is a threat of ocular perforation [54]. Surgical procedures include debridement or lamellar keratectomy, formation of a conjunctival flap over a severely ulcerated area of the cornea (in an attempt to save the eyeball), or penetrating keratoplasty if a donor cornea is available. The goals of the therapeutic penetrating keratoplasty (TPK) are to primarily eliminate the infection and restore the integrity of the globe. The cure rate of TPK for fungal keratitis varies from 60 to 90% with a recurrence rate of 6–15% [55]. In intractable cases, with perforation of the eye, evisceration is required [6]. In low-resource settings, the availability of specialist ophthalmologists able to perform such surgeries is limited. More studies are required to prove the value and timing of surgical intervention. The development of collaborative, local training programs are needed to address these training needs.

## 6. Prevention

Certain public health measures may reduce the incidence of fungal keratitis and improve outcomes. Public health campaigns in localities with a high incidence encourage rapid self-referral to the hospital for early diagnosis and treatment to achieve better outcomes. Education of primary healthcare workers on the clinical signs and symptoms of corneal infection may also increase early recognition and prompt referral [8]. Studies from Asia have demonstrated the efficacy of prompt prophylactic treatment of corneal abrasions with chloramphenicol and clotrimazole eye drops in preventing the development of microbial keratitis [56,57,58]. One initiative in Bhutan trained village health workers to identify post-traumatic corneal abrasions with fluorescein dye and a blue light torch [57]. Moreover, the introduction of protective glasses in agricultural workers and manual labourers would likely reduce the incidence of ocular trauma, which leaves individuals susceptible to fungal infection. Education of contact lens wearers on proper, hygienic practices may be useful. Much more work is needed to develop such effective public health strategies and assess their value.

## 7. Conclusions

Fungal keratitis is a severe and disabling disorder that, if diagnosed promptly, is usually responsive to current therapies. The designation of NTD status will engage countries most heavily burdened, leading to the implementation of prevention and awareness programmes. NTD status will prompt the education of community health workers, in addition to ophthalmologists, on the early signs and symptoms of fungal keratitis, and this will increase early recognition and treatment. Finally, it will spur the development of more effective antifungal agents and better availability of existing medications. Ultimately, NTD designation will likely decrease the incidence of this serious disease and improve the prognosis of those affected.

## Data Availability

Not applicable.
